# Comparison of GPT-4o With Human Performance in the Polish Vascular Surgery Specialty Examination

**DOI:** 10.7759/cureus.93022

**Published:** 2025-09-23

**Authors:** Michalina Loson-Kawalec, Anna Kowalczyk, Aleksander Tabor, Patrycja Dadynska, Aleksandra Wielochowska, Dawid Boczkowski, Tomasz Dolata, Weronika Majchrowicz, Piotr Sawina, Dominika Radej, Maja Kruplewicz, Dawid Bartosik, Marta Zerek, Alicja Szalach, Gracjan Sitarek, Ada Latkowska

**Affiliations:** 1 Medicine, University of Opole, Opole, POL; 2 Internal Medicine, Karol Marcinkowski University Hospital, Zielona Góra, POL; 3 Internal Medicine, Central Teaching Hospital of the Medical University of Lodz, Lodz, POL; 4 Internal Medicine, Multispecialty Independent Public Health Care Institution Hospital in Nowa Sól, Nowa Sól, POL; 5 Internal Medicine, Non-Public Health Care Institution (NZOZ) Hospital in Dzierżoniów, Dzierżoniów, POL; 6 General Surgery, Department of General and Vascular Surgery, Specialist Medical Center, Polanica-Zdrój, POL; 7 Dentistry, Dental Office - Dentistry in the Tenement, Gryfino , POL; 8 Medicine, Wroclaw Medical University, Wrocław, POL

**Keywords:** artificial intelligence(ai), chat gpt-4o, medical education, pes, vascular surgery

## Abstract

Background

Artificial intelligence (AI) offers many possibilities by using language models such as ChatGPT, also known as a synthetic generative intelligence chatbot advanced by OpenAI (OpenAI, Inc., San Francisco, USA). Using the potential of AI in medicine can provide a crucial tool to assess medical expertise and create a promising future in the field of medical education. Prior investigations have documented the progressive advancing performance of AI systems in addressing medical situations. These studies were also conducted in the evaluation of Polish medical examinations, comprising the State Specialization Examination (PES) as discussed in this article. These findings have stimulated scholarly debate regarding the potential of such technologies to serve as instruments for enhancing postgraduate specialist education and training.

Objective

This study aimed to evaluate the performance of the ChatGPT-4o model in solving the PES in the field of vascular surgery. The analysis examined both the correctness of the answers and the model’s stated confidence, with the goal of understanding its potential value in education.

Methods

This study was developed using the official PES in vascular surgery from a previous session, namely, the Spring 2025 edition, comprising 120 multiple-choice items. The ChatGPT-4o model was acquainted with the examination regulations beforehand, and all items were presented in the Polish language. Response accuracy was evaluated against the database of correct answers of the Medical Examination Center (CEM) in Łódź and also included the model’s self-reported confidence rating on a five-point scale. Statistical analyses were conducted using the chi-square test to compare categorical variables and the Mann-Whitney U test to assess differences between non-normally distributed continuous variables.

Results

ChatGPT-4o achieved 88 correct answers (73.3%), thereby surpassing the minimum passing criterion for the examination. There was no apparent distinction in the efficacy of clinical and non-clinical questions (p=0.561). The model's self-reported confidence levels did not largely correlate with its response accuracy. Such discrepancies show that, while ChatGPT can imply its doubts, it is not able to consistently predict performance, highlighting ongoing limitations in the model’s self-assessment capabilities.

Conclusions

ChatGPT-4o demonstrated satisfactory results on the PES vascular surgery exam, highlighting AI’s promise in specialist education, particularly as a support for learning a special field of medicine with specific conditions. It is crucial to treat ChatGPT as a supporting educational tool, not exclusively used by one source of knowledge. These findings indicate that advanced AI models may serve as valuable tools in a specialist field of education. Nonetheless, careful oversight by medical professionals and additional validation studies across various medical fields are necessary before AI models can be widely implemented in medical education.

## Introduction

Artificial intelligence (AI), when combined with relevant tools and knowledge, has a profound impact on the advancement of medicine. This solution offers numerous benefits and has the potential to yield substantial improvements in healthcare. The introduction of AI is set to transform both medical education content and improve patient care, medical research, and health systems [1]. The integration of AI into medicine is generating widespread fascination. AI's ability to process and analyze enormous volumes of data, quickly identify patterns and signals, and precisely predict results is superior to that of humans. Hopes are high that AI-driven healthcare will lead to greater precision and efficiency while also reducing costs for both patients and health systems.

At the same time, AI is a source of concern. Key ethical issues include questions of trust, privacy, bias, accountability, and responsibility. To address these challenges, various efforts are underway to create ethical frameworks aimed at preventing AI from unleashing unforeseen negative consequences. Both national and international initiatives are working to establish principles and safeguards to ensure the responsible use of AI [2]. The artificial intelligence behind ChatGPT is transforming a wide range of industries. According to the *Financial Times*, OpenAI’s tools are used by around 500 million people each week [3].

It has been four years since OpenAI (San Francisco, USA) launched the first version of ChatGPT. During this time, the company has introduced five major releases, along with numerous updates and sub-versions. These analyses included a series of models spanning recent releases: GPT-3.5 (2022), GPT-4 (2023), GPT-4 with tool integration (2023), GPT-4 Turbo (2023), and the GPT-4o, launched in May 2024. This model acquires any combination of text, audio, and image as input and generates any of their combinations. GPT-4o is capable of processing speech, sound, images, and text in real time, while being faster and more cost-efficient than GPT-4 Turbo. It also provides improved multimodal context understanding, emotion recognition, interpretation of images and charts, and supports voice-based interaction.

With the advancement of AI, there is a growing global interest in applying modern AI models in various areas of medicine. In a 2023 study, Kufel et al. found that a previous version of ChatGPT failed to pass the Polish State Specialization Exam (PES) in radiology [4]. Jaworski et al. in May 2024 showed that, after being acquainted with the examination regulations, ChatGPT-4o could reach a passing score on the Medical-Dental Final Examination [5]. A similar occurrence was reported later, in July 2025, when B. Sławinska et al. achieved high effectiveness on the PES ophthalmology examination [6]. 

The dynamic evolution of AI, illustrated by OpenAI’s models, highlights the need for continuous monitoring. Responding to rapid changes requires systematic research that captures and reports progress in their development and adaptation into the realities of healthcare operations.

The objective of this study was to assess the performance of the ChatGPT-4o language model on questions from the PES vascular surgery examination, with emphasis on the accuracy of its answers and its self-reported confidence levels. The evaluation was conducted using the official correct answers database of the Medical Examination Center (CEM) in Łódź and by comparing it with the answers generated by ChatGPT [7].

## Materials and methods

This study used the GPT-4o model and was performed from the 12th of August to the 18th of August 2025. The study focused on one vascular surgery specialized test from the Spring 2025 that was selected at random from the CEM in Łódź's archive database. The examination questions and their official answers are publicly accessible and readily available. The chosen test had 120 questions with five distractions, each of which had only one right response. The Examination Board did not rule out any of the 120 questions, indicating that they were all in line with what was currently known.

For analysis, the examination questions were grouped into two types: those based on clinical cases and those addressing non-clinical topics. Clinical case questions described patient scenarios and required responses based on clinical reasoning, symptom analysis, test result interpretation, and decisions regarding diagnostic and therapeutic procedures. The questions in the non-clinical group assessed theoretical understanding, treatment recommendations, or factual data unrelated to a particular patient case. Question classification was conducted independently by two researchers, with any disagreements resolved by a third, independent reviewer. Before encountering the exam questions, ChatGPT was briefed on the exam format, including the total questions, answer choices, and correct answers.

The answers provided were systematically evaluated according to the official database of the correct answers, which was released by CEM. For analysis, all questions and the corresponding answers were systematically compiled and documented. After each question was posed, ChatGPT was asked to assign a confidence score from 1 to 5, where 1 indicated no confidence, 2 low, 3 moderate, 4 high, and 5 complete confidence. This method provided a measure of the model’s self-evaluated certainty for each response.

ChatGPT was shown every exam question, and every exchange was methodically documented. To ensure consistency with the PES vascular surgery exam content, all communications with the model were conducted in Polish. Microsoft Excel (Microsoft® Corp., Redmond, USA) program and GraphPad Prism 10 (GraphPad Software Inc., Boston, USA) were used to conduct statistical analyses. To evaluate the model’s performance, differences in confidence ratings for correct versus incorrect responses were analyzed using the Mann-Whitney U test, while the chi-square test was applied to compare the frequency of correct and incorrect answers across clinical and non-clinical question categories. P-values below 0.05 were regarded as statistically significant.

Given the multicenter design of the study, collaboration among authors was conducted via remote communication platforms, including Microsoft Teams (Microsoft® Corp., Redmond, USA), email, and Google Docs (Google, Inc., Mountain View, USA). Contributions prepared by individual teams were reviewed by other authors, ensuring that all researchers were able to participate in and provide input on every section of the manuscript through these remote tools.

## Results

**Figure 1:**
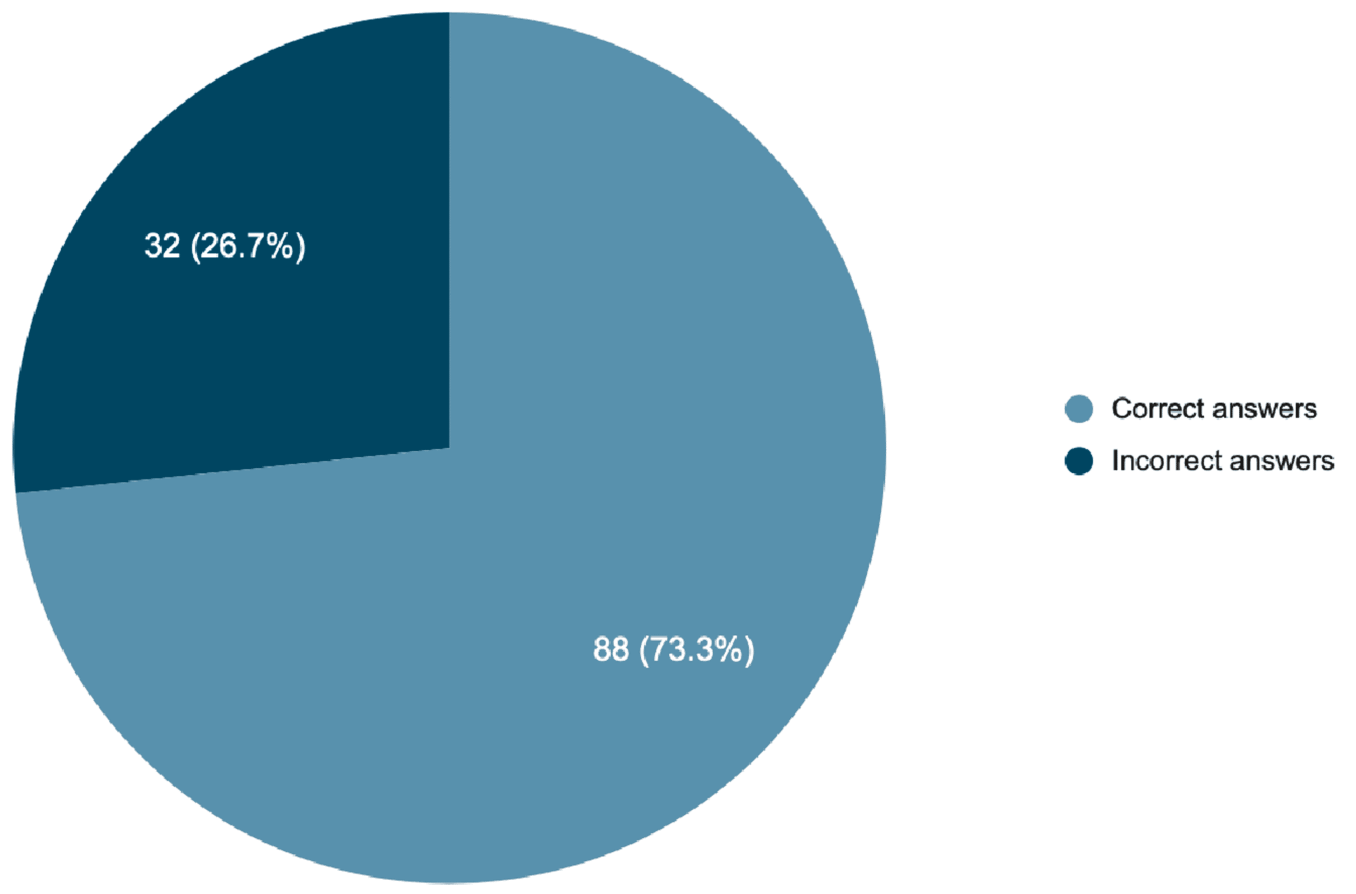
General summary of GPT-4o The results are reported as the number of responses (N) along with their corresponding percentage of the total questions (%), indicating the proportion of correct or incorrect answers.

**Figure 2:**
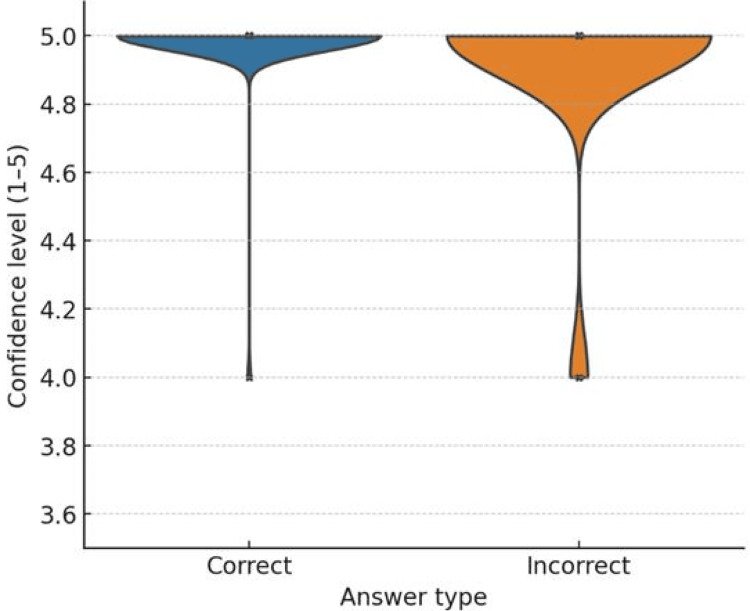
Effectiveness of ChatGPT-4o depending on the question type: clinical cases vs. others Values are presented individually for correct and incorrect responses. No significant association was found between confidence levels and correct answers. The connection between response accuracy and the confidence ratings provided by ChatGPT was assessed using the Mann–Whitney U test.

ChatGPT-4o answered 88 questions correctly (73.3%) and 32 incorrectly (26.7%) (Figure 1, Table 1). No statistically significant difference in accuracy was observed between clinical and non-clinical questions (χ² = 0.337, p = 0.561) (Table 2, Figure 2). No strong association was observed between confidence ratings and the correctness of responses, indicating that, in this context, the model’s self-reported confidence was not a reliable predictor of answer accuracy (U = 1336; p > 0.001) (see Appendices).

**Table 1:** Comparison of ChatGPT-4o responses to PES vascular surgery exam questions and the model’s reported confidence levels This table summarizes the model’s answers in relation to the correct responses as specified by the official correct answers database published by the CEM in Łódź. For each question, the model’s self-rated confidence is provided on a five-point scale, ranging from 1 (not confident at all) to 5 (completely confident). PES: Polish State Specialization Exam; CEM: Medical Examination Centre

Question number	Official answer	ChatGPT answer	ChatGPT confidence level (1-5)
1	C	B	5
2	E	E	4
3	A	A	5
4	B	B	5
5	E	E	5
6	D	A	5
7	E	E	5
8	A	A	5
9	D	E	5
10	A	D	5
11	A	A	5
12	C	C	5
13	A	A	5
14	D	D	5
15	D	D	5
16	B	B	5
17	D	B	5
18	E	B	5
19	C	C	5
20	A	A	5
21	B	D	5
22	C	C	5
23	E	B	5
24	D	D	5
25	A	A	5
26	C	C	5
27	B	B	5
28	C	C	5
29	C	C	5
30	C	C	5
31	D	D	5
32	E	E	5
33	A	A	5
34	D	B	4
35	C	C	5
36	A	A	5
37	E	E	5
38	E	E	5
39	C	C	5
40	C	C	5
41	A	A	5
42	C	C	5
43	A	A	5
44	C	C	5
45	D	D	5
46	E	E	5
47	B	B	5
48	B	A	5
49	C	C	5
50	B	D	5
51	B	B	5
52	B	B	5
53	A	C	5
54	B	B	5
55	C	C	5
56	B	B	5
57	D	D	5
58	B	B	5
59	C	C	5
60	C	C	5
61	B	B	5
62	E	C	4
63	B	B	5
64	D	D	5
65	B	B	5
66	D	A	5
67	B	B	5
68	A	E	5
69	A	A	5
70	B	A	5
71	D	D	5
72	A	B	5
73	E	D	5
74	B	C	5
75	C	C	5
76	B	B	5
77	B	B	5
78	E	E	5
79	D	D	5
80	D	C	5
81	A	E	5
82	B	B	5
83	E	E	5
84	E	E	5
85	C	C	5
86	D	D	5
87	D	C	5
88	D	D	5
89	C	D	5
90	C	C	5
91	A	A	5
92	D	D	5
93	B	B	5
94	C	E	5
95	C	C	5
96	B	A	5
97	A	A	5
98	B	E	5
99	A	A	5
100	C	B	5
101	B	B	5
102	D	D	5
103	D	D	5
104	E	B	5
105	B	D	5
106	C	C	5
107	A	A	5
108	C	C	5
109	B	B	5
110	C	C	5
111	D	D	5
112	B	A	5
113	D	D	5
114	E	D	5
115	B	B	5
116	A	A	5
117	D	B	5
118	E	C	5
119	D	D	5
120	E	E	5

**Table 2:** Effectiveness of ChatGPT-4o depending on the question type: clinical cases vs. others Data are presented as absolute numbers (N) and percentages (%). Comparison between the groups revealed no statistically significant differences (p = 0.561; χ² = 0.337).

Type of question	Correct answers, n (%)	Incorrect answers, n (%)	p-Value χ² Value
Clinical cases	47 (39.17)	19 (15.83)	p = 0.561 χ² = 0.337
Other	41 (34.17)	13 (10.83)	

## Discussion

The PES exam is a crucial step in qualifying a physician as a specialist in a specific medical specialty. The PES exam is considered passed when the physician achieves a score of at least 60% of the maximum possible score. It is known for being extremely challenging and requiring both theoretical understanding and real-world problem-solving abilities in clinical settings. This study focuses on the way the sophisticated ChatGPT-4o language model performs on tasks related to vascular surgical exams. In similar investigations in other medical domains, the model's pass score is higher than scores attained by previous iterations of ChatGPT models and surpasses the pass standards. Currently, no other studies in the literature have examined ChatGPT’s performance on the vascular surgery PES exam.

Recent advances in AI models, specifically the debut of ChatGPT-4o, have led to a notable improvement in performance, reflecting progress in the medical field’s understanding of such systems. In our investigation, the accuracy of the model’s replies was not largely correlated with its confidence level. 

These results indicate that the model is, at least partially, capable of evaluating its own performance and expressing doubts. However, the model is not able to consistently predict performance, highlighting ongoing limitations in the model’s self-assessment capabilities. Importantly, these differences were not statistically significant according to the Mann-Whitney U test. While emphasizing the potential of AI as a valuable resource in medical education, the consistency of these conclusions across different examinations also emphasizes the need for careful application under the proper expert supervision. Additionally, our results might be comparable to a study on Japan's National Physical Therapist Examination [8]. Our study's findings also confirm the effectiveness and high efficiency of using GPT-4.0 for questions entered in a native language, suggesting its usefulness for healthcare professionals who do not speak English.

Crucially, when contrasting our current findings with the previously mentioned study [8], it becomes clear that ChatGPT-4o's frequent use of assertive language may be misleading, even if it is inaccurate, particularly in crucial industries, including healthcare. The success rate of ChatGPT-4.0 in this study was 73.4%, which is roughly comparable to the vascular surgery score of 73.3%. The model exceeded the 60% pass mark in both cases, indicating its educational applicability in advanced specialist examinations. A notable similarity between the two studies lies in the model’s accuracy in answering questions based on clinical expertise. Additionally, the authors of this study reported that GPT-4's performance on clinical cases with images and tables is lower, indicating areas for improvement.

The effectiveness of different ChatGPT versions in medical examinations has also been investigated, particularly in the fields of infectious diseases [9], dermatology [10], and allergology [11]. Our findings reveal a comparable trend: the model’s confidence level did not show significant differences between correct or incorrect answers, similar to performance on clinical questions, which do not differ significantly from other question types. In reference to these results, the use of language models in medical education still presents certain limitations. In medical contexts, such models may generate responses that appear plausible but are, in fact, inaccurate, potentially leading to undesirable conclusions. Therefore, with appropriate academic supervision, their role in case studies, training, and education should be regarded as supportive rather than definitive.

There are several significant limitations to this study. Due to the absence of previous research examining the application of language models in this field, it is based solely on a single Polish specialist examination in vascular surgery. The generalizability of these results to other countries or international specialist exams may be limited by variations in exam format, language, and regional medical practices. Opportunities for comparison with the existing literature are inherently limited. The probabilistic nature of the model's responses, which can produce variable results across multiple trials, limits the study's reproducibility. Furthermore, if ChatGPT-4o's training data included material overlapping with the examination content, performance scores may have been artificially inflated.

## Conclusions

The ChatGPT-4o model resulted in a positive pass rate and demonstrated successful performance in addressing PES vascular surgery exam questions from the Spring 2025 session, achieving results above the passing threshold. These findings indicate that advanced AI models may serve as valuable tools in specialist education, particularly for self-assessment, exam simulation, and knowledge testing under realistic conditions. Despite exceeding the pass mark, the GPT-4o chat model failed to demonstrate reliability, as it overestimated its capabilities in incorrect answers. This suggests that artificial intelligence cannot currently be relied upon as a sole source of information or as an assistance in medical practice.

ChatGPT-4o may also function as an interactive teaching assistant, facilitating education by recognizing learning deficiencies and supplying customized feedback, due to its capacity to generate human-like responses and self-assess confidence. Although early findings suggest that language models can be of assistance in diagnostic testing and exam preparation, long-term comparative studies across various medical professions remain in short supply. The integration of such systems into medical education necessitates further investigation, both to validate their effectiveness across different medical specialties and to establish safe, ethical, and practical implementation frameworks. Moreover, the development of clear standards for expert oversight and the definition of boundaries for AI-assisted learning are essential to ensure that these technologies enhance education without supplanting critical thinking or professional judgment.

## Appendices

**Table 3:** Questions and responses of the ChatGPT-4o model in the Polish vascular surgery specialty examination

Question	Chat Answer	Confidence Level
Question 1: Indicate the false statement regarding venous claudication: A. pain in the thigh occurs during vigorous physical exertion of the lower limb. B. clinical symptoms limit physical fitness only to a very small extent. C. the cause of swelling and pain is valvular insufficiency and reflux in the deep veins of the lower limb. D. the cause of clinical symptoms is a high, constant outflow resistance in the collateral veins. E. it occurs in patients with chronic occlusion of the iliac-femoral segment veins.	B	5/5
Question 2: Classic recommendations regarding indications for repair of femoral artery aneurysms include: 1) all symptomatic aneurysms of any size; 2) aneurysms with mural thrombus; 3) aneurysms with a diameter not less than 25 mm; 4) aneurysms that enlarge during follow-up; 5) aneurysms in which the initial pulse examination result has changed, indicating embolism. The correct answer is: A.1,2. B.1,3. C.1,3,4. D.3,4,5. E.all of the above.	E	4/5
Question 3: When performing an incision along the anterior edge of the sartorius muscle to obtain medial access to the proximal segment of the popliteal artery, one should be cautious because of the anatomical proximity of: 1) the great saphenous vein; 2) the small saphenous vein; 3) the saphenous nerve; 4) the common peroneal nerve; 5) the medial sural cutaneous nerve. The correct answer is: A.1,3. B.1,4. C.2,5. D.1,5. E.all of the above.	A	5/5
Question 4: A round vascular catheter with a size of 2F has an external diameter of: A.0.2 mm. B.2/3 mm. C.1 mm. D.3/2 mm. E.2 mm.	B	5/5
Question 5: An indication for an extra-anatomic femoro-femoral bypass is not: A.infection of a previously implanted graft at the site of the planned anatomical bypass. B.severe general condition of the patient, making it impossible to safely perform the operation in a traditional manner. C.advanced age, especially in debilitated patients. D.anticipated great technical difficulties in performing the operation by the traditional method. E.bilateral atherosclerosis of the femoral arteries.	E	5/5
Question 6: An indication for immediate surgery of an aortic aneurysm is: 1) symptomatic aneurysm; 2) aneurysm with a diameter exceeding 5.5 cm; 3) rupture of the aneurysm; 4) infectious aneurysm; 5) embolizing aneurysm. The correct answer is: A.1,3. B.1,2,3,5. C.3,4,5. D.only 3. E.1,2,4,5.	A	5/5
Question 7: The radiological criterion that does not indicate functional intestinal ischemia is: A.narrowing of many branches of the SMA. B.“sausage string” sign of intestinal loops. C.spasm of the mesenteric arcades. D.abnormal filling of vessels in the intestinal wall. E.dissected intimal flap of the SMA.	E	5/5
Question 8: One of the early complications after implantation of a stent graft into an abdominal aortic aneurysm may be the occurrence of a post-implantation syndrome. Indicate the true statement regarding this syndrome: A.it resolves spontaneously after 7–10 days. B.it requires intravenous antibiotic therapy with cephalosporins. C.its occurrence is a predictive factor for endoleak. D.it is associated with contrast-induced nephropathy. E.it is a consequence of postoperative peripheral ischemia of the lower limbs.	A	5/5
Question 9: Symptoms of stenosis or occlusion of the internal carotid artery do not include: A.motor and sensory disturbances on the side opposite to the stenosis. B.speech disturbances if the stenosis is on the side of the dominant hemisphere. C.visual disturbances on the side of the stenosis. D.tinnitus. E.loss of consciousness and fainting.	E	5/5
Question 10: The most common complication after resection of a carotid body tumor is injury to the nerve: A.vagus (X). B.glossopharyngeal (IX). C.facial (VII). D.answers A and B are correct. E.answers A, B, and C are correct.	D	5/5
Question 11: The most common location of embolic material in acute intestinal ischemia is: A.the SMA segment distal to the origin of the middle colic artery. B.the SMA segment distal to the origin of the right colic artery. C.the SMA segment distal to the origin of the ileocolic artery. D.the region of the SMA origin. E.the region of the IMA origin.	A	5/5
Question 12: A 45-year-old patient, 5 years after creation of a radiocephalic fistula, developed dialysis disturbances. A strong thrill was observed over the fistula in the distal forearm, and a weak bruit in the proximal forearm. Control ultrasound showed 65% stenosis of the left cephalic vein. In this situation, the recommended treatment is: A.conservative – monthly ultrasound monitoring of the fistula. B.surgical – classic reconstruction with removal of the stenosis. C.surgical – percutaneous endovascular angioplasty, access via the cephalic vein. D.surgical – access via the brachial artery. E.surgical – access via the femoral artery.	C	5/5
Question 13: In a 75-year-old patient on dialysis using a brachio-basilic fistula, pathological dilation of the fistula with a palpable thrill was observed. Proximally, a relatively good bruit was present. Ultrasound revealed brachial artery blood flow of about 2000 ml/min, anastomosis width of 6 mm, followed proximally by dilation of the basilic vein to 2.5 cm over 10 cm, a stenosis about 1 cm long with a minimum diameter of 3 mm, and a straight segment about 8 mm in diameter and 5 cm in length. In this situation, the appropriate management is: A.reconstruction of the fistula – aneurysmoplasty of the dilated segment combined with endovascular angioplasty of the stenotic segment, with the fistula being usable for dialysis immediately. B.reconstruction of the fistula – aneurysmoplasty of the dilated segment combined with endovascular angioplasty of the stenotic segment, but the remaining fistula is too short for immediate dialysis use, so a dialysis catheter is necessary. C.reconstruction of the fistula – aneurysmoplasty of the dilated segment, with the fistula being usable for dialysis immediately. D.reconstruction of the fistula – removal of the dilated segment and stenosis using a PTFE graft, with the unreconstructed part of the fistula suitable for immediate dialysis. E.conservative treatment with monthly ultrasound control of the fistula.	A	5/5
Question 14: In a 75-year-old patient previously dialyzed using catheters through the right and then the left internal jugular vein, an arteriovenous brachiocephalic fistula was created on the left arm. In the follow-up examination 4 weeks after surgery, a rather weak bruit was heard over the fistula without palpable thrill. Ultrasound revealed an increase in cephalic vein diameter from 3 mm to 3.5 mm and brachial artery blood flow from 20 ml/min to 150 ml/min. In this situation, one should conclude: A.after 2 weeks, start cannulation of the dialysis fistula. B.use a hybrid dialysis method with single-needle cannulation for blood return and a catheter for blood withdrawal for 3 weeks to accelerate fistula maturation, then remove the catheter. C.use a hybrid dialysis method with single-needle cannulation for blood withdrawal and a catheter for blood return for 3 weeks to accelerate fistula maturation, then remove the catheter. D.the fistula is not maturing properly, most likely due to insufficient inflow. E.the fistula is not maturing properly, most likely due to outflow obstruction.	D	5/5
Question 15: In a 75-year-old patient, 4 years after creation of a radiocephalic fistula, a 75% stenosis about 2 cm proximal to the anastomosis caused dialysis disturbances. Percutaneous endovascular balloon angioplasty was performed with balloon inflation for 2 minutes at 10 atm. Control angiography revealed a residual stenosis of about 40%. In this situation, the next step is: A.remove the endovascular equipment, considering the stenosis adequately corrected. B.remove the endovascular equipment and perform classic fistula reconstruction. C.inflate the balloon again at 10 atm for 2 minutes at the stenosis site. D.inflate the balloon again at the stenosis site to at least 20 atm. E.use a paclitaxel-coated balloon.	D	5/5
Question 16: In a 45-year-old patient, a radiocephalic fistula was created 3 years earlier and has been used for 2 years without problems. During the last 3 dialysis sessions, alarms related to decreased arterial line pressure were noted. The patient self-reported weakening bruit and thrill over the fistula. The probable cause is: A.outflow disturbance from the fistula caused by stenosis in the draining vein. B.inflow disturbance caused most likely by stenosis at or near the arteriovenous anastomosis. C.suboptimal cannulation technique of the fistula. D.fistula maturation causing its gradual degradation. E.increased inflow to the fistula related to its maturation.	B	5/5
Question 17: In a 45-year-old patient, previously a manual worker, after starting dialysis via a permanent dialysis catheter inserted through the right internal jugular vein, a radiocephalic fistula was created on the left forearm. After 3 weeks, the cephalic vein was dilated to 6 mm, blood flow was 600 ml/min, and vein wall thickness was 0.05 mm. In this situation, the correct management is: A.remove the dialysis catheter and start cannulation of the fistula. B.use a hybrid dialysis method for 3 weeks with single-needle cannulation of the fistula for blood withdrawal and blood return via the catheter to accelerate fistula maturation, then remove the catheter. C.use a hybrid dialysis method for 3 weeks with single-needle cannulation of the fistula for blood return and blood withdrawal via the catheter to accelerate fistula maturation, then remove the catheter. D.wait another 3 weeks for the fistula to mature, dialyzing via the catheter in this period, and after several successful cannulations qualify the patient for catheter removal. E.parameters indicate fistula maturation disorder, corrective procedures are necessary.	B	5/5
Question 18: A 45-year-old patient with end-stage renal disease and diabetes reported right hand pain and blanching of the index finger, symptoms worsening during hemodialysis using a brachiocephalic fistula on the right side. With the fistula functioning, oxygen saturation measured on the right index finger was 88%, increasing to 91% after temporary compression of the fistula. Ultrasound revealed fistula blood flow about 600 ml/min, brachial artery flow distal to the anastomosis about 20 ml/min, and cephalic vein diameter about 7 mm. In this situation, the management is: A.ligate the fistula. B.narrow the fistula under control of blood flow volume. C.refer the patient to a neurologist for treatment of neurological disorders. D.refer the patient to a diabetologist for microangiopathy. E.perform arteriography and conduct vascular treatment based on its findings.	B	5/5
Question 19: The presence of a good systolic-diastolic bruit over the initial segment of a radiocephalic fistula after compression of the cephalic vein about 15 cm proximal to the anastomosis indicates: A.good inflow to the fistula and correct outflow via a single venous trunk. B.good inflow to the fistula and the presence of a large collateral proximal to the site of fistula compression. C.good inflow to the fistula and the presence of a large collateral between the anastomosis and the site of compression. D.poor inflow to the fistula and good outflow. E.poor inflow to the fistula and poor outflow.	C	5/5
Question 20: In a 45-year-old patient, a brachiocephalic fistula was created 3 years earlier and used for 2 years without problems. During the last 3 dialysis sessions, high venous line pressures, increased recirculation, and problems with hemostasis after removal of both needles were observed. The probable cause is: A.outflow disturbance from the fistula caused by stenosis in the cephalic arch. B.inflow disturbance caused by stenosis at the arteriovenous anastomosis. C.suboptimal cannulation technique of the fistula. D.fistula maturation causing its gradual degradation. E.increased inflow to the fistula related to its maturation.	A	5/5
Question 21: A 24-year-old woman with an impaired left ventricular ejection fraction (20%), with a cardioverter defibrillator implanted through the left subclavian vein, due to progressive renal failure is qualified for urgent initiation of renal replacement therapy with hemodialysis. In such a situation, the optimal dialysis access for this patient is: A. a permanent dialysis catheter inserted through the right internal jugular vein. B. a permanent dialysis catheter inserted through the right femoral vein. C. a radiocephalic fistula in the left upper limb. D. a radiocephalic fistula in the right upper limb. E. a brachiocephalic fistula in the right upper limb.	D	5/5
Question 22: Antibiotic prophylaxis in the treatment of abdominal aortic aneurysm: A. is not required. B. is required only in open surgery of the aneurysm. C. is required in both open and endovascular surgery of the aneurysm. D. does not reduce the risk of infection. E. none of the above.	C	5/5
Question 23: Before surgery of an abdominal aortic aneurysm (AAA), one should: A. always perform a control Doppler ultrasound of the carotid arteries. B. in the case of internal carotid artery stenosis greater than 70%, first perform revascularization. C. every patient should undergo coronary angiography. D. taking statins has no effect on aneurysm treatment outcomes. E. none of the above.	B	5/5
Question 24: Indicate the false statement regarding visceral artery aneurysms: A. A symptom of a hepatic artery aneurysm may be jaundice. B. A visceral artery aneurysm that occurs almost twice as often in women as in men is a splenic artery aneurysm. C. A celiac trunk aneurysm most often develops on the basis of medial degeneration. D. A splenic artery aneurysm should always be treated in elderly patients, because the risk of rupture and death on this basis is greatest. E. Chronic pancreatitis is an important risk factor for the development of a gastroduodenal artery aneurysm.	D	5/5
Question 25: Endovascular repair of a popliteal artery aneurysm has been successfully practiced since 1994. Indicate the true statement: A. The minimum proximal and distal landing zone is 1.5 cm. B. The endovascular method shows a higher long-term patency rate compared to classical femoropopliteal bypass. C. It is the recommended method in acute limb ischemia due to thrombosis of a popliteal aneurysm. D. Similarly to endovascular repair of an abdominal aortic aneurysm, it does not require DAP in the postoperative period. E. Due to the lower risk of tibial nerve injury, the endovascular method is recommended in young, physically and professionally active people.	A	5/5
Question 26: The most common cause of abdominal angina symptoms is: A. Occlusion of the inferior mesenteric artery. B. Ostial stenosis of the celiac trunk. C. Ostial stenosis of the superior mesenteric artery. D. Distal occlusion of the superior mesenteric artery. E. None of the above.	C	5/5
Question 27: Tessari’s technique describes: A. A technique of distal limb revascularization with a dialysis fistula with simultaneous ligation at the A-V anastomosis. B. A technique for creating foam for sclerotherapy. C. Endovascular reconstruction of the aortic bifurcation using stent grafts. D. Creation of an arteriovenous fistula for hemodialysis using a HeRO graft. E. Performing retrograde access in endovascular recanalization of calf vessels in patients with diabetic foot syndrome.	B	5/5
Question 28: Indicate the false statement regarding superficial thrombophlebitis: A. The basis of treatment is early mobilization of the patient, the use of NSAIDs and compression therapy. B. In the case of thrombosis with the thrombus head near the deep system outlet (<3 cm), treatment should be implemented as for DVT. C. In the case of the need for low-molecular-weight heparin at a dose of 40 mg, treatment should last 30 days. D. Ablation or removal of the great saphenous vein should be considered in patients with symptoms of superficial thrombophlebitis and concomitant reflux in ultrasound. E. The use of NSAIDs reduces the risk of extension of superficial thrombophlebitis, but without reducing the risk of pulmonary embolism.	C	5/5
Question 29: Minimally invasive non-thermal methods of treatment of insufficient superficial veins do NOT include: A. Mechanochemical ablation. B. Cyanoacrylate glue ablation. C. Laser ablation. D. The use of an occluder with additional sclerotherapy. E. All of the listed methods are non-thermal minimally invasive methods of treating insufficient superficial veins.	C	5/5
Question 30: Symptoms of the “nutcracker syndrome” include: A. Hematuria, abdominal pain radiating to the right hypochondrium, proteinuria. B. Abdominal pain, glucosuria, right-sided varicocele. C. Abdominal pain, hematuria, proteinuria. D. Vulvar varices, abdominal pain radiating to the right hypochondrium, proteinuria. E. Right-sided varicocele, proteinuria, hematuria.	C	5/5
Question 31: External iliac artery endofibrosis most often affects young men practicing competitive sports. The sport discipline not predisposing to the development of external iliac artery stenosis in this mechanism is: A. Cycling. B. Triathlon. C. Rowing. D. Swimming. E. Cross-country skiing.	D	5/5
Question 32: A vascular hemorrhagic diathesis is: A. von Willebrand disease. B. Glanzmann’s thrombasthenia. C. Bernard-Soulier syndrome. D. Disseminated intravascular coagulation (DIC). E. Henoch-Schönlein purpura.	E	5/5
Question 33: In the diagnosis of antiphospholipid syndrome, clinical criteria (thrombotic and obstetric complications) and laboratory criteria (confirmed twice at least 12 weeks apart presence of lupus anticoagulant/anticardiolipin antibodies/anti-β2 glycoprotein I antibodies) are distinguished. To diagnose antiphospholipid syndrome, it is sufficient to have at least: A. One clinical criterion and one laboratory criterion. B. One clinical criterion and two laboratory criteria. C. Two laboratory criteria. D. Two clinical criteria (one thrombotic and one obstetric) and one laboratory criterion. E. Two clinical criteria (one thrombotic and one obstetric).	A	5/5
Question 34: Cerebral hyperperfusion syndrome after recanalization of the internal carotid artery is diagnosed when cerebral blood flow increases by: A. 25%. B. 50%. C. 75%. D. 100% or more. E. Cerebral hyperperfusion syndrome always occurs after recanalization of the carotid artery regardless of the degree of cerebral blood flow increase.	B	4/5
Question 35: Lymphangiosarcoma developing in long-term lymphedema is: A. Meige’s disease. B. Milroy’s disease. C. Stewart-Treves syndrome. D. Harjola-Marabala syndrome. E. May-Thurner syndrome.	C	5/5
Question 36: Milroy’s disease is associated with a mutation of the gene encoding the receptor for: A. Vascular endothelial growth factor (VEGF). B. Fibroblast growth factor (FGF). C. Insulin-like growth factor (IGF). D. Transforming growth factor (TGF). E. Epidermal growth factor (EGF).	A	5/5
Question 37: Cystic adventitial degeneration occurs more often in men and is the cause of intermittent claudication in 1:1200 patients with lower limb ischemia. In 80% of cases, the pathology involves the artery: A. Common iliac artery. B. External iliac artery. C. Common femoral artery. D. Superficial femoral artery. E. Popliteal artery.	E	5/5
Question 38: A vascular compression syndrome is NOT: A. Dunbar syndrome. B. Harjola-Marabala syndrome. C. May-Thurner syndrome. D. Nutcracker syndrome. E. Milroy’s disease.	E	5/5
Question 39: The arterial thoracic outlet syndrome is characterized by the following symptoms: 1) Aneurysmal degeneration of the subclavian artery; 2) Acute embolism in the upper limb; 3) Chronic embolism in the upper limb; 4) Thrombosis of the subclavian artery; 5) Hypertrophy of the hand muscles. The correct answer is: A. 1,2,4. B. 1,3,4. C. 1,2,3,4. D. 1,3,4,5. E. 2,3,5.	C	5/5
Question 40: Which type of endoleak after endovascular repair of an abdominal aortic aneurysm qualifies absolutely for corrective treatment? 1) Leak at the aortic or iliac attachment sites of the graft; 2) Patent vessels originating from the aneurysm sac; 3) Defect of the graft material or disconnection of stent graft modules; 4) Leak between fibers of the graft material; 5) In every case of endoleak corrective treatment should be undertaken. The correct answer is: A. Only 1. B. 2,4. C. 1,3. D. 1,4. E. Only 5.	C	5/5
Question 41: To prevent the growth of a thrombus in acute limb ischemia caused by arterial embolism, one should administer: A. Unfractionated heparin at a dose of 80 IU/kg body weight. B. Enoxaparin sodium 2 × 40 mg. C. Alteplase 100 mg. D. Streptokinase 250,000 IU. E. Acetylsalicylic acid 160–325 mg/24 h.	A	5/5
Question 42: After carotid endarterectomy, symptoms of cerebral ischemia occurred on the first postoperative day. Ultrasound showed no flow through the internal carotid artery. What should be the correct management? A. Local thrombolysis. B. Conservative treatment and, if symptoms persist, reoperation after 24 hours. C. Immediate reoperation and restoration of flow. D. PTA with stent implantation. E. Conservative treatment.	C	5/5
Question 43: The following statements concern the technique of treating acute mesenteric ischemia due to mesenteric artery thrombosis. The technique combining surgery and endovascular treatment is: A. Retrograde open mesenteric stenting. B. Endarterectomy. C. Bypass using the patient’s own vein. D. Stenting via puncture of the brachial artery. E. Embolectomy.	A	5/5
Question 44: Indicate the true statements regarding the technique of recanalization of chronic total iliocaval occlusions: 1) it is best to use a catheter with a profiled tip and a hydrophilic coating; 2) perforations during attempts to cross the occluded segment may cause the patient to bleed to death; 3) technically the guidewire should be passed under the venous intima; 4) often a long sheath 45–55 cm is required for additional rigid support for the guidewire. The correct answer is: A. 1,2. B. 1,3. C. 1,4. D. 2,3. E. 3,4.	C	5/5
Question 45: Indicate the true statements regarding the technique of venous stenting: 1) balloon angioplasty alone of the iliac veins is very often sufficient without the need for stent placement; 2) venous stents should be large in caliber, similar to the natural lumen of the vessel; 3) stents in the iliac veins should cover the entire diseased segment, not just the critical stenosis; 4) extensive placement of stents in the veins increases the likelihood of thrombosis. The correct answer is: A. 1,2. B. 1,3. C. 1,4. D. 2,3. E. 3,4.	D	5/5
Question 46: Ischemic rest pain in the classification of chronic limb ischemia corresponds to: A. Stage IIb according to Fontaine and stage 3 according to Rutherford. B. Stage IIb according to Fontaine and stage 4 according to Rutherford. C. Stage III according to Fontaine and stage 3 according to Rutherford. D. Stage III according to Fontaine and stage 5 according to Rutherford. E. Stage III according to Fontaine and stage 4 according to Rutherford.	E	5/5
Question 47: The basic method of symptomatic treatment of intermittent claudication is: A. Chelation. B. Physical exercise, walking training. C. Cilostazol. D. Acetylsalicylic acid. E. Combined anticoagulant treatment with ASA and a low dose of rivaroxaban.	B	5/5
Question 48: A patient with ischemic diabetic foot and soft tissue necrosis was qualified for amputation at the level of the Chopart joint. Indicate the correct anatomical name of this bone structure: A. Tarsometatarsal joint. B. Transverse tarsal joint. C. Metatarsophalangeal joint. D. Talocrural joint. E. Interphalangeal joint.	A	5/5
Question 49: The most optimal primary vascular access for dialysis is an arteriovenous fistula: A. Brachial-ulnar. B. Brachial-radial. C. Radial-radial. D. Radial-ulnar. E. Ulnar-ulnar.	C	5/5
Question 50: During Doppler ultrasonography in a patient with chronic venous insufficiency, the presence of the Giacomini vein was found. This indicates a connection between the following veins: A. Small saphenous and popliteal. B. Small saphenous and great saphenous. C. Great saphenous and femoral. D. Great saphenous and popliteal. E. Great saphenous and superficial circumflex iliac.	D	5/5
Question 51: In the treatment of which type of endoleak into the sac of an abdominal aortic aneurysm after stent graft implantation is access through the so-called arc of Riolan most often used? A. I. B. II. C. III. D. IV. E. V.	B	5/5
Question 52: Which of the measurements obtained from computed tomography angiography is the least important when planning the implantation of a bifurcated stent graft for an infrarenal abdominal aortic aneurysm (AAA)? A. Length of the common iliac arteries. B. Length of the external iliac arteries. C. Length of the neck of the AAA. D. Diameter of the neck of the AAA. E. Diameter of the external iliac arteries.	B	5/5
Question 53: Many vascular surgeons, during open surgery of a popliteal artery aneurysm, often choose the posterior approach with the patient lying prone. Which of the following anatomical structures is usually not present in this area? A. Saphenous nerve. B. Sural nerve. C. Fibular (peroneal) nerve. D. Tibial nerve. E. Small saphenous vein.	C	5/5
Question 54: In a 65-year-old patient, computed tomography angiography revealed May-Thurner syndrome. This means compression of: A. The left renal vein by the superior mesenteric artery. B. The left common iliac vein by the right common iliac artery. C. The subclavian vein by the first rib. D. The inferior vena cava by an abdominal aortic aneurysm. E. The popliteal vein by the head of the gastrocnemius muscle.	B.	5/5
Question 55: Which type of endoleak occurs most frequently after implantation of a bifurcated stent graft for an abdominal aortic aneurysm? A. IA. B. IB. C. II. D. III. E. IV.	C	5/5
Question 56: In a 70-year-old patient, several hours after embolectomy due to acute lower limb ischemia, massive swelling of the calf muscles occurred, requiring fasciotomy. In which fascial compartment does compression of the anterior tibial nerve and artery most often occur? A. Lateral fibular. B. Anterior superficial. C. Posterior deep. D. Posterior superficial. E. None of the above.	B	5/5
Question 57: What values of intra-abdominal pressure (IAP) allow the diagnosis of abdominal compartment syndrome after implantation of a stent graft for a ruptured infrarenal abdominal aortic aneurysm? A. Above 5 mmHg. B. Above 10 mmHg. C. Above 15 mmHg. D. Above 20 mmHg. E. None of the above.	D	5/5
Question 58: Which artery supplies the carotid body tumor most significantly? A. Common carotid. B. External carotid. C. Internal carotid. D. Superior thyroid. E. Branches of the thyrocervical trunk.	B	5/5
Question 59: Indicate the true statements regarding the classification WIfI introduced by the European Society for Vascular Surgery: 1) it is used to assess the risk of limb amputation in patients with chronic limb-threatening ischemia; 2) it allows assessment of the potential benefit of revascularization; 3) it considers the extent of tissue loss and the severity of infection; 4) it considers ABI values when assessing limb perfusion; 5) it considers the distance of intermittent claudication. The correct answer is: A. 1,3,5. B. 2,3,4,5. C. 1,2,3,4. D. 1,4,5. E. All of the above.	C	5/5
Question 60: Which Doppler ultrasound finding indicates the presence of hemodynamically significant renal artery stenosis? A. Peak systolic velocity (RA-PSV) between 0.5–0.9 m/s. B. Peak systolic velocity (RA-PSV) between 1.0–1.8 m/s. C. Aorto-renal ratio above 3.5. D. Aorto-renal ratio below 3.5. E. Reversal of flow in the branches of the renal artery.	C	5/5
Question 61: Indicate the statement correctly describing the so-called bovine aortic arch seen on computed tomography angiography: A. Common origin of both subclavian arteries from the aortic arch. B. Common origin of the brachiocephalic trunk and the left common carotid artery from the aortic arch. C. Separate origin of the right subclavian artery from the aortic arch below the brachiocephalic trunk. D. Separate origin of the right common carotid artery from the aortic arch. E. Separate origin of the right subclavian artery from the aortic arch below the left subclavian artery.	B	5/5
Question 62: Based on the current classification, which type of blood leakage into the abdominal aortic aneurysm sac occurring after stent graft implantation is relatively the most difficult to visualize in follow-up spiral computed tomography: A. IA. B. IB. C. II. D. III. E. IV.	C	4/5
Question 63: The surgical treatment of celiac trunk compression syndrome consists of cutting: A. the gastrocolic ligament and reimplantation of the hepatic artery into the aorta above the stenosed celiac trunk. B. the median arcuate ligament of the diaphragm and fibers of the celiac plexus. C. the gastrocolic ligament and reimplantation of the splenic artery into the aorta above the stenosed celiac trunk. D. the hepatogastric ligament and fibers of the celiac plexus. E. the crura of the diaphragm.	B	5/5
Question 64: The finding of a positive Stemmer’s sign on clinical examination is typical for edema of: A. after revascularization procedures on lower limb arteries. B. associated with arteriovenous malformations. C. lipedema. D. lymphatic origin. E. venous origin.	D	5/5
Question 65: In a 70-year-old female patient with diabetic nephropathy, six months after the creation of a brachiocephalic arteriovenous fistula for dialysis, symptoms of finger ischemia developed. The consulting vascular surgeon recommended performing a DRIL operation, which should consist of: A. placement of a banding on the arterialized cephalic vein. B. construction of a bypass with the saphenous vein and ligation of the distal brachial artery segment below the vascular anastomosis. C. construction of a bypass with the saphenous vein without ligation of the distal brachial artery segment below the vascular anastomosis. D. embolization of cephalic vein collaterals. E. implantation of a self-expanding stent into the primary vascular anastomosis.	B	5/5
Question 66: In a 60-year-old patient with end-stage renal failure, an arteriovenous fistula for dialysis was created from a subcutaneously transposed basilic vein in the arm. Which neural structure runs in direct proximity to this vessel? A. ulnar nerve. B. median nerve. C. lateral cutaneous nerve of the forearm. D. medial cutaneous nerve of the forearm. E. radial nerve.	A	5/5
Question 67: In endovascular surgery of thoracic aortic aneurysms, the landing zone of the stent graft that includes the initial 2 cm beyond the origin of the left subclavian artery is designated by the number: A. 1. B. 3. C. 5. D. 7. E. 9.	B	5/5
Question 68: Indicate the false statements concerning Loeys-Dietz syndrome: 1) aneurysm formation occurs at an older age than in Marfan syndrome; 2) symptoms include, among others, blue sclera, joint hypermobility, and skin hyperextensibility; 3) the main cause of death is dissection of the ascending aorta; 4) surgical qualification guidelines include, among others, a subrenal aortic diameter greater than 4 cm; 5) conservative treatment includes, among others, angiotensin receptor blockers. The correct answer is: A.1,2. B.1,3,4. C.1,2,3,5. D.2,3,5. E.1,2,4.	E	5/5
Question 69: The key structures lying above the bifurcation of the common carotid artery are: 1) posterior belly of the digastric muscle; 2) hypoglossal nerve; 3) veins running from the sternocleidomastoid muscle to the internal jugular vein; 4) small arteries supplying the neck muscles arising from the posterior branches of the internal carotid artery; 5) vestibulocochlear nerve. The correct answer is: A.1,2,3. B.2,3,4. C.tylko 2. D.1,2,3,4. E.1,3,4,5.	A	5/5
Question 70: According to the TASC II classification of femoropopliteal lesions, a single popliteal stenosis is classified as type: A. A. B. B. C. C. D. D. E. E.	A	5/5
Question 71: Indicate the true statements concerning Raynaud’s syndrome: 1) symptoms affect fingers and toes equally; 2) an attack begins with pallor, then cyanosis, and finally redness of the fingers, and symptoms persist between attacks; 3) the most common trigger is exposure to low temperature; 4) the vasospastic type of Raynaud’s syndrome is characterized by significant narrowing or occlusion of digital arteries; 5) the most commonly used drugs in treatment are alpha-blockers. The correct answer is: A.1,2,3. B.2,3,5. C.2,4. D.3. E. none of the above.	D	5/5
Question 72: Infection of a vascular prosthesis is most often caused by: A. Staphylococcus aureus. B. Staphylococcus epidermidis. C. Proteus mirabilis. D. Escherichia coli. E. fungi.	B	5/5
Question 73: An indication for revascularization of the left subclavian artery during stent graft implantation into the thoracic aorta with coverage of its origin in patients with non-penetrating aortic injuries is NOT: A. patent left internal mammary artery for coronary bypass grafting. B. absence, small size, or occlusion of the right vertebral artery. C. planned coverage of ≥20 cm of the descending thoracic aorta at the origin of intercostal arteries. D. occlusion of the hypogastric artery. E. all of the above are indications for revascularization.	D	5/5
Question 74: The most common cause of subclavian artery aneurysm formation is: A. trauma. B. thoracic outlet syndrome. C. atherosclerosis. D. iatrogenic complications. E. fibromuscular dysplasia.	C	5/5
Question 75: Indicate the false statement concerning the treatment of a popliteal artery aneurysm: A. surgical treatment is possible through medial or posterior access. B. posterior access is not possible if the aneurysm involves the superficial femoral artery or arteries below the knee. C. medial access allows full decompression of the aneurysm, which is not always possible with posterior access. D. endovascular treatment (stent graft implantation) is possible if outflow is preserved through at least 2 calf vessels. E. endovascular treatment (stent graft implantation) is possible if proximal and distal landing zones of 15–20 mm are preserved.	C	5/5
Question 76: Indicate the false statement concerning thoracic outlet syndrome (TOS): A. one cause may be the presence of a cervical rib, although its presence does not necessarily cause symptoms. B. because the brachial plexus and subclavian vein pass through the scalene triangle, compression usually involves both of these structures, and the venous and neurogenic forms of TOS may be confused. C. one of the clinical tests used in TOS diagnosis is Adson’s test, which involves assessment of the radial artery pulse during abduction of the limb with head rotation and breath-holding. D. in the neurogenic form of TOS, conservative treatment is initially indicated, and surgery should be used if there is no response to less invasive methods. E. in surgical treatment, the axillary approach allows resection of the first rib but only partial scalenotomy.	B	5/5
Question 77: Indicate the false statement concerning the use of protamine sulfate: A. it neutralizes low-molecular-weight heparins less effectively than unfractionated heparin. B. 1 mg of protamine sulfate neutralizes 50 IU of unfractionated heparin. C. its dosage depends on the amount of unfractionated heparin administered within the last 2 hours. D. its effectiveness can be assessed by measuring aPTT. E. rapid administration of protamine sulfate may cause hypotension and bradycardia.	B	5/5
Question 78: In the case of lack of proper maturation of a dialysis fistula due to blood steal from the main trunk of the fistula into its side branches, treatment may consist of: A. surgical ligation of the side branch through a small incision. B. subcutaneous puncture of the side branch. C. endovascular embolization of the side branch with coils. D. endovascular embolization of the side branch with cyanoacrylate glue. E. all of the above.	E	5/5
Question 79: What is the preferred location for implantation of a dialysis catheter? A. non-tunneled catheter – left internal jugular vein, tunneled catheter – right internal jugular vein. B. non-tunneled catheter – right femoral vein, tunneled catheter – right subclavian vein. C. both non-tunneled and tunneled catheters – right or left femoral vein. D. both non-tunneled and tunneled catheters – right internal jugular vein. E. both non-tunneled and tunneled catheters – right or left subclavian vein.	D	5/5
Question 80: Indicate the true statement concerning the assessment of dialysis fistula maturation using ultrasound: A. blood flow should be 200 ml/min and vein diameter 4 mm, with depth not exceeding 4 mm. B. blood flow should be 400 ml/min and vein diameter 4 mm, with depth not exceeding 4 mm. C. blood flow should be 400 ml/min and vein diameter 4 mm, with depth not exceeding 6 mm. D. blood flow should be 600 ml/min and vein diameter 6 mm, with depth not exceeding 6 mm. E. blood flow should be 600 ml/min and vein diameter 4 mm, with depth not exceeding 6 mm.	C	5/5
Question 81: Indicate the true statement regarding the measurement of internal carotid artery stenosis according to NASCET and ECST: A. Both methods use measurement of the residual lumen diameter at the stenosis. B. Both methods use measurement of the normal internal carotid artery diameter. C. NASCET uses measurement of the estimated carotid bulb diameter. D. ECST uses measurement of the normal internal carotid artery diameter. E. Differences in assessing stenosis are most evident in very high-grade stenoses.	E	5/5
Question 82: One complication of internal carotid endarterectomy may be nerve injury. Indicate the true statement: A. Most commonly the glossopharyngeal nerve is injured, causing loss of swallowing ability. B. Most commonly the hypoglossal nerve is injured, causing tongue deviation toward the injured side. C. Most commonly a branch of the facial nerve is injured, causing lower lip paralysis. D. Most commonly the hypoglossal nerve is injured, causing tongue deviation to the opposite side. E. Most commonly the vagus nerve is injured, causing hoarseness.	B	5/5
Question 83: The type of atherosclerotic plaque in which ruptures, hematomas, or thrombi occur is: A. I B. III C. IV D. V E. VI	E	5/5
Question 84: Indicate the false statement regarding popliteal artery entrapment syndrome: A. Results from abnormal embryological development of the popliteal artery and gastrocnemius muscle. B. MRI is the gold standard in imaging. C. Type 1 is most common, with the artery displaced toward the normally positioned medial head of the gastrocnemius. D. Type 6 is functional entrapment with symptoms but no identifiable anatomical anomaly. E. Treatment is always surgical, requiring vascular bypass in every case.	E	5/5
Question 85: Indicate the false statement regarding compartment syndrome: A. Occurs when tissue pressure in a closed anatomical space rises enough to impair blood flow. B. Symptoms include firmness and tenderness of the compartment, paresthesias in nerves supplied by the compartment, and muscle weakness. C. In the lower leg, it most commonly occurs in the lateral compartment. D. Percutaneous measurement of compartment pressure is a diagnostic method. E. Surgical decompression is recommended when compartment pressure is ≥40 mmHg.	C	5/5
Question 86: Indicate the false statement regarding non-penetrating thoracic aortic injuries: A. Grade II refers to intramural hematoma. B. Grade III refers to pseudoaneurysm. C. Blood pressure–lowering pharmacotherapy is important to reduce rupture risk. D. Routine revascularization of the left subclavian artery is indicated when covered by a stent graft. E. Routine cerebrospinal fluid drainage is not recommended in endovascular treatment.	D	5/5
Question 87: In the grading scale of non-penetrating carotid artery injuries, artery occlusion corresponds to: A. I B. II C. III D. IV E. V	C	5/5
Question 88: Indicate the false statement regarding extracranial carotid artery aneurysms: A. Most commonly occur in the common carotid artery, least in the external carotid artery. B. Presence of an aneurysm indicates repair regardless of symptoms. C. Internal carotid aneurysm repair may involve excision, artery mobilization, and primary end-to-end anastomosis without graft. D. After excision of external carotid aneurysm, vascular reconstruction is always required. E. Internal carotid aneurysm repair may involve excision and end-to-end anastomosis between proximal external carotid and distal internal carotid.	D	5/5
Question 89: Indicate the true statements regarding visceral artery aneurysms: 1) Bleeding from hepatic artery aneurysms occurs equally into the peritoneal cavity and biliary tract. 2) Bleeding from gastric or gastroepiploic aneurysms occurs more often into the peritoneal cavity than the GI tract. 3) Endovascular treatment is preferred for hepatic artery aneurysms. 4) Jejunal branch aneurysms rupture more frequently than ileal branch aneurysms. 5) Endovascular treatment is preferred for splenic artery aneurysms. The correct answer is: A. 1,2,3,4 B. 1,3,4,5 C. 1,3,5 D. 2,3,5 E. 2,4,5	D	5/5
Question 90: Indicate the true statements regarding devices used in atherectomy: 1) Orbital, directional, laser, and rotational atherectomy devices are used. 2) Directional and rotational devices require embolic protection. 3) Orbital atherectomy can be performed via true lumen or subintimal technique. 4) Directional atherectomy is contraindicated subintimally. 5) A specific complication of orbital atherectomy is hemolysis, causing hemoglobinuria or hypertensive crisis. The correct answer is: A.1,2,3,5 B.1,2,4,5 C.1,2,5 D.2,3,4 E.3,4	C	5/5
Question 91: Compared with balloon-expandable stents, self-expanding stents have: A. Lower radial force. B. Lower flexibility. C. Greater placement precision. D. Both A and B. E. A, B, and C.	A	5/5
Question 92: Indicate the false statement regarding vitamin K antagonists (VKA): A. Full anticoagulant effect develops after 4–5 days. B. Antibiotics can enhance VKA effect. C. Low albumin increases VKA effect; high albumin decreases it. D. Most common adverse effect is bleeding, usually in the urinary tract. E. In case of bleeding: stop VKA, give vitamin K, transfuse FFP or PCC.	D	5/5
Question 93: Indicate the true statements regarding dialysis catheters: 1) Non-tunneled catheters are used if dialysis is planned for >2 weeks. 2) Tunneled catheters have lower infection rates. 3) Subclavian vein access has the highest infection rate. 4) If standard access sites are unavailable, lumbar vein access to the IVC is an alternative. 5) Femoral vein catheters should end in the external iliac vein. The correct answer is: A.1,2,4 B.2,4 C.2,3,5 D.1,4,5 E. Only 2	B	5/5
Question 94: Indicate the false statements regarding dialysis fistula assessment and monitoring: 1) Fistula murmur should cover the cardiac cycle; its absence may indicate thrombosis. 2) Augmentation test confirms and localizes a large steal branch. 3) Segmental occlusion test detects stenosis at fistula origin. 4) Elevating the limb causes collapse in a well-functioning native fistula. 5) In symptomatic steal syndrome, compressing the fistula worsens ischemia. The correct answer is: A.1,2,3 B.1,3,4 C.2,3,5 D.2,4,5 E.2,3,4,5	E	5/5
Question 95: Indicate the true statement regarding vertebral artery reconstruction: A. Vascular reconstruction is performed at all vertebral artery levels. B. Proximal vertebral artery disease is treated by transposing it to the contralateral common carotid. C. For distal vertebral artery revascularization, a bypass from the common carotid using autologous vein is possible. D. Proximal vertebral artery revascularization uses anastomosis with external carotid. E. Distal vertebral artery revascularization can be done by transposition to the subclavian artery.	C	5/5
Question 96: When assessing vessel diameter for dialysis fistula creation by ultrasound, standard qualification requires: A. Arteries ≥2 mm, veins ≥4 mm, and prosthetic veins ≥6 mm. B. Arteries ≥2 mm, veins ≥2 mm, prosthetic veins ≥4 mm. C. Arteries ≥3 mm, veins ≥3 mm, prosthetic veins ≥6 mm. D. Arteries ≥4 mm, veins ≥4 mm, prosthetic veins ≥6 mm. E. Arteries ≥4 mm, veins ≥2 mm, prosthetic veins ≥4 mm.	A	5/5
Question 97: Indicate the true statement regarding neuroprotection devices used during carotid stenting: A. Using a distal filter may require pre-dilation with a balloon. B. Distal filter does not allow angiographic assessment during the procedure. C. Proximal neuroprotection requires crossing the stenosis. D. Proximal device requires smaller sheath than distal filter. E. Removing distal filter does not require passing it through the implanted stent.	A	5/5
Question 98: Which is not a criterion for high-risk carotid endarterectomy patients: A. Age >80 years B. Highly tortuous aortic arch and great vessels C. Left ventricular ejection fraction <30% D. Contralateral vocal cord paralysis E. Occlusion of the contralateral carotid artery	E	5/5
Question 99: Unusual (“exotic”) upper limb dialysis fistulas include: 1) Ulnar-ulnar 2) Brachio-brachial 3) Brachio-ulnar 4) Radial-ulnar 5) Brachio-radial A.1,2,4 B.1,2,3 C.2,3,5 D.2,4,5 E.1,3,5	C	5/5
Question 100: Which phenomenon is NOT observed on Doppler ultrasound in hemodynamically significant ICA stenosis: A. Decreased resistance in the ipsilateral external carotid artery. B. Reduction in systolic and diastolic velocity distal to ICA stenosis. C. Decreased resistance proximal to ICA stenosis. D. Increased flow velocity in contralateral internal carotid artery. E. Increased flow velocity in contralateral vertebral artery.	B	5/5
Question 101: One of the risk stratification scales for thromboembolic complications in general surgery patients is: A. CEAP B. Caprini C. Wagner D. Puig E. Barthel	B	5/5
Question 102: In suspected or diagnosed HIT, UFH and LMWH should be stopped. All HIT patients should be treated with which anticoagulant: A. Enoxaparin B. Nadroparin C. Dalteparin D. Fondaparinux E. Acenocoumarol	D	5/5
Question 103: In case of high suspicion of HIT, you perform: A. Protein C level B. Protein S level C. Antithrombin III level D. Anti-heparin/PF4 antibody test E. APTT correction test	D	5/5
Question 104: For reconstruction of a short segment of the common femoral or iliac vein, which materials can be used: 1) PTFE graft 10–14 mm 2) Autologous great saphenous vein 3) Autologous superficial femoral vein 4) Autologous external jugular vein 5) Autologous internal jugular vein The correct answer is: A. only 1 B. 1,2 C. 1,2,3 D. 2,3,4 E. all	B	5/5
Question 105: De Palma procedure: 1) Balloon-expandable stent for iliac artery occlusion 2) Surgery for symptomatic unilateral iliac vein occlusion 3) Suprapubic femoral vein transposition to decompress venous obstruction 4) If no suitable vein, suprapubic PTFE 6–8 mm graft 5) If no suitable vein, suprapubic Dacron 6–8 mm graft The correct answer is: A. only 1 B. only 2 C. 2,3 D. 2,3,4 E. 2,3,5	D	5/5
Question 106: Horner syndrome as a complication of intraoperative sympathetic fiber injury during carotid artery surgery manifests as: 1) Ptosis on the affected side 2) Miosis on the affected side 3) Iris heterochromia darker on affected side 4) Anhidrosis on affected side 5) Cutaneous vasoconstriction on affected side The correct answer is: A. 1,2,3 B. 1,2,3,4 C. 1,2,4 D. 1,2,3,5 E. 3,4,5	C	5/5
Question 107: Screening for congenital thrombophilia is not indicated: A. Young patients with provoked proximal DVT being considered for stopping anticoagulation B. Young patients with unprovoked proximal DVT being considered for stopping anticoagulation C. Patient with unprovoked proximal DVT and strong family history D. DVT in atypical locations E. B and C	A	5/5
Question 108: Pharmacological treatment after carotid endarterectomy (CEA) includes: A. LMWH for 1 month B. Aspirin + clopidogrel C. Aspirin + statins D. Iloprost E. No antiplatelet therapy needed	C	5/5
Question 109: During duplex Doppler of the popliteal artery, normal flow is observed. According to Polish Society of Vascular Surgery, this flow is called: A. Low-resistance B. High-resistance C. Continuous D. Biphasic E. Monophasic	B	5/5
Question 110: Diameter of a stent graft for AAA relative to aneurysm neck should be: A. Equal B. 5–10% larger C. 10–20% larger D. 20–30% larger E. 30–40% larger	C	5/5
Question 111: A patient with acute abdomen on chronic anticoagulation (AF) is brought to the ED. Last dose unknown. For which NOACs does APTT have no prognostic value for intraoperative bleeding risk: 1) Apixaban 2) Dabigatran 3) Edoxaban 4) Rivaroxaban 5) Warfarin The correct answer is: A. 1,2,3,4 B. 2,4,5 C. 1,2,4 D. 1,3,4 E. 1,5	D	5/5
Question 112: Ability to exclude DVT using D-dimer depends on clinical probability (Wells score). A highly specific test can exclude DVT in patients with: A. Only low probability B. Low and moderate probability C. Low, moderate, and high probability D. Moderate and high probability E. Only high probability	A	5/5
Question 113: Causes of early intraoperative spinal cord ischemia include: A. Blockage of perfusion via superior mesenteric artery and possible Riolan arch closure B. Intravascular thrombosis C. Decreased cardiac output due to arrhythmia from low intracellular potassium D. Decreased spinal cord perfusion pressure E. Undetected left-to-right atrial shunt	D	5/5
Question 114: Contraindications to cilostazol: 1) Congestive heart failure 2) MI in last 6 months 3) Proliferative diabetic retinopathy 4) Creatinine clearance ≤25 ml/min 5) Concomitant use of ≥2 antiplatelet or anticoagulant drugs The correct answer is: A.1,3 B.1,3,5 C.3,4,5 D.1,2,4 E. all	D	5/5
Question 115: Most common visceral artery aneurysm: A. Celiac trunk B. Splenic artery C. Superior mesenteric artery D. Renal artery E. Common hepatic artery	B	5/5
Question 116: “Refolding of valves” technique refers to: A. Longitudinal internal valvuloplasty (Kistner) B. Internal T-shaped valvuloplasty (Sattiuari) C. Excisional technique D. External valvuloplasty (Nicolaides/Kistner/Rayu/Hoshino/Gloviczki) E. None of the above	A	5/5
Question 117: During carotid-subclavian transposition for proximal left subclavian artery occlusion, surgical access requires: A. Cut sternocleidomastoid and platysma B. Retract anterior and middle scalene muscles to expose proximal subclavian artery C. Always preserve thoracic duct at right venous angle D. Preserve phrenic nerve on anterior scalene E. Preserve phrenic nerve on middle scalene	B	5/5
Question 118: Recommended inter-surface compression pressure for venous ulcer therapy (American Venous Forum): A. 18–21 mmHg B. 23–32 mmHg C. 30–35 mmHg D. 32–40 mmHg E. >40 mmHg	C	5/5
Question 119: High diaphragm on radiograph after supraclavicular approach to subclavian artery indicates: A. Pneumonia B. Pneumothorax and pleural injury C. Pleural effusion and bleeding D. Phrenic nerve injury E. Thoracic duct injury	D	5/5
Question 120: Characteristic clinical finding NOT seen in chronic mesenteric ischemia: A. Postprandial abdominal pain 15–45 min after meals B. Weight loss C. Often associated with abdominal aortic aneurysm D. Nausea, diarrhea E. Fasting abdominal pain in morning	E	5/5
